# Understanding the Role of Adenosine A2AR Heteroreceptor Complexes in Neurodegeneration and Neuroinflammation

**DOI:** 10.3389/fnins.2018.00043

**Published:** 2018-02-06

**Authors:** Dasiel O. Borroto-Escuela, Sonja Hinz, Gemma Navarro, Rafael Franco, Christa E. Müller, Kjell Fuxe

**Affiliations:** ^1^Department of Neuroscience, Karolinska Institutet, Stockholm, Sweden; ^2^Section of Physiology, Department of Biomolecular Science, University of Urbino, Campus Scientifico Enrico Mattei, Urbino, Italy; ^3^Observatorio Cubano de Neurociencias, Grupo Bohío-Estudio, Yaguajay, Cuba; ^4^PharmaCenter Bonn, Pharmaceutical Institute, Pharmaceutical Chemistry I, University of Bonn, Bonn, Germany; ^5^Department of Biochemistry and Molecular Biology, Faculty of Biology, University of Barcelona, Barcelona, Spain

**Keywords:** G protein-coupled receptor, neurodegeneration, adenosine A2A receptor, heteroreceptor complexes, oligomerization, adenosine receptor, Parkinson's diseases, neuroinflammation

## Abstract

Adenosine is a nucleoside mainly formed by degradation of ATP, located intracellularly or extracellularly, and acts as a neuromodulator. It operates as a volume transmission signal through diffusion and flow in the extracellular space to modulate the activity of both glial cells and neurons. The effects of adenosine are mediated via four adenosine receptor subtypes: A1R, A2AR, A2BR, A3R. The A2AR has a wide-spread distribution but it is especially enriched in the ventral and dorsal striatum where it is mainly located in the striato-pallidal GABA neurons at a synaptic and extrasynaptic location. A number of A2AR heteroreceptor complexes exist in the striatum. The existence of A2AR-D2R heteroreceptor complexes with antagonistic A2AR-D2R interactions in the striato-pallidal GABA neurons is well-known with A2AR activation inhibiting Gi/o mediated signaling of D2Rs. A2AR-mGluR5 heteroreceptor complexes were also found in with synergistic receptor-receptor interactions enhancing the inhibition of the D2R protomer signaling. They are located mainly in extrasynaptic regions of the striato-pallidal GABA neurons. Results recently demonstrated the existence of brain A2AR-A2BR heteroreceptor complexes, in which A2BR protomer constitutively inhibited the function of the A2AR protomer. These adenosine A2AR heteroreceptor complexes may modulate alpha-synuclein aggregation and toxicity through postulated bidirectional direct interactions leading to marked increases in A2AR signaling both in nerve cells and microglia. It is of high interest that formation of A2AR-A2ABR heteroreceptor complexes provides a brake on A2AR recognition and signaling opening up a novel strategy for treatment of A2AR mediated neurodegeneration.

## Introduction

Adenosine is a nucleoside mainly formed by degradation of ATP located intracellularly or extracellularly and acts as a neuromodulator (Fredholm, 1995; Gomes et al., 2011). In the extracellular fluid of the neuronal-glial networks of the Central Nervous System (CNS), it operates as a volume transmission signal through diffusion and flow to modulate the activity of both glial cells and neurons (Fuxe et al., 2010). It also acts as a homeostatic modulator (Gomes et al., 2011). Intracellular adenosine can reach the extracellular space through inter alia equilibrative transporters in the plasma membrane. The effects of adenosine are mediated via four adenosine receptor subtypes: A1R, A2AR, A2BR, A3R (Fredholm, 1995; Fredholm et al., 2011).

The A1R is distributed all over the CNS and found in high densities. It is coupled to G_i/o_ and inter alia inhibits adenylyl cyclase (AC) and calcium channels, and activates potassium channels. Being located both in synaptic and extrasynaptic positions at the pre and postsynaptic level, it inhibits synaptic transmission and hyperpolarizes nerve cells (Gomes et al., 2011). The A1R is also expressed in astroglia and microglia to fine tune neuronal-glial interactions.

The A2AR has a wide-spread distribution but it is especially enriched in the ventral and dorsal striatum where it is mainly located in the striato-pallidal GABA neurons at a synaptic and extrasynaptic location (Fuxe et al., 2003; Gomes et al., 2011; Brugarolas et al., 2014; Navarro et al., 2016a). It is coupled to G_s/olf_ proteins, activates AC, enhances glutamate release and enhances the activity of the striato-pallidal GABA neurons involving also inhibition of their inhibitory dopamine D2R located at the postsynaptic level (Fuxe et al., 2007; Gomes et al., 2011).

Unlike the A2AR the A2BR is coupled G_s/q_, which leads to PLC activation and increases in intracellular calcium levels (Linden et al., 1999; Fredholm et al., 2011; Goncalves et al., 2015). The A2BR has been scarcely studied in the CNS. A difference from A1R and A2AR is that they are activated by adenosine only at micromolar adenosine concentrations, which may be only reached in pathological states (Müller and Stein, 1996). Their physiological role remains unclear but highly interesting results were recently obtained demonstrating the existence of A2AR-A2BR heteroreceptor complexes, in which A2BR protomer constitutively inhibited the function of the A2AR protomer (Hinz et al., 2017, in press).

With regard to the A3R it was shown to be located in neurons of the hippocampus based on the presence of A3R mRNA levels in hippocampal neurons using single cell PCR analysis (Lopes et al., 2003). The A3R possesses a high affinity for adenosine and is mainly coupled to G_i2.3._ It therefore inhibits AC signaling but it can also activate PLC (Fredholm et al., 2011). Activation of A3Rs found in hippocampal nerve terminal membranes using Western blot analysis, produces neuroprotective actions (Boison, 2010; Boison and Shen, 2010; Fishman et al., 2012). It is of high interest that A3R activation selectively brings down persistent pain states through its analgesic properties (Little et al., 2015).

In the current perspective paper we will give an update of the adenosine A2AR heteroreceptor complexes that may be expressed in the CNS and to which extent they may participate and help explain molecular mechanisms underlying the involvement of adenosine receptors in neurodegeneration and neuroinflammation.

## Adenosine A2ARs modulate alpha-synuclein aggregation and toxicity. possible involvement of A2AR heteroreceptor complexes

### Adenosine A2A receptor-alpha-synuclein interactions

Alpha-synuclein mediated excitotoxicity involves increased calcium flow over the NMDA channels (Diogenes et al., 2012). In an exciting paper Ferreira et al. (2017a) demonstrated that A2AR antagonists and A2AR genetic deletion counteracted cell death produced by alpha-synuclein. The number of cells forming inclusions of alpha-synuclein was also reduced but not oligomerization of alpha-synuclein. In contrast, A2AR activation enhanced calcium flow over the NMDA channels (Rebola et al., 2008) as found after alpha-synuclein. Furthermore, the toxic effects of alpha-synuclein on long term depression (LTD) were abolished by reducing A2AR activity. It is of high interest that this rescue of synaptic function was dependent on NMDA receptor signaling (Ferreira et al., 2017a,b). In line with these results it was suggested that activation of A2ARs can produce a substantial increase of activity in synaptic NMDA receptor function causing excitotoxicity (Besancon et al., 2008). Aberrant A2A receptor signaling demonstrated in synucleinopathy participates in reductions of cognition and neurodegeneration (Hu et al., 2016).

**Figure 1:**
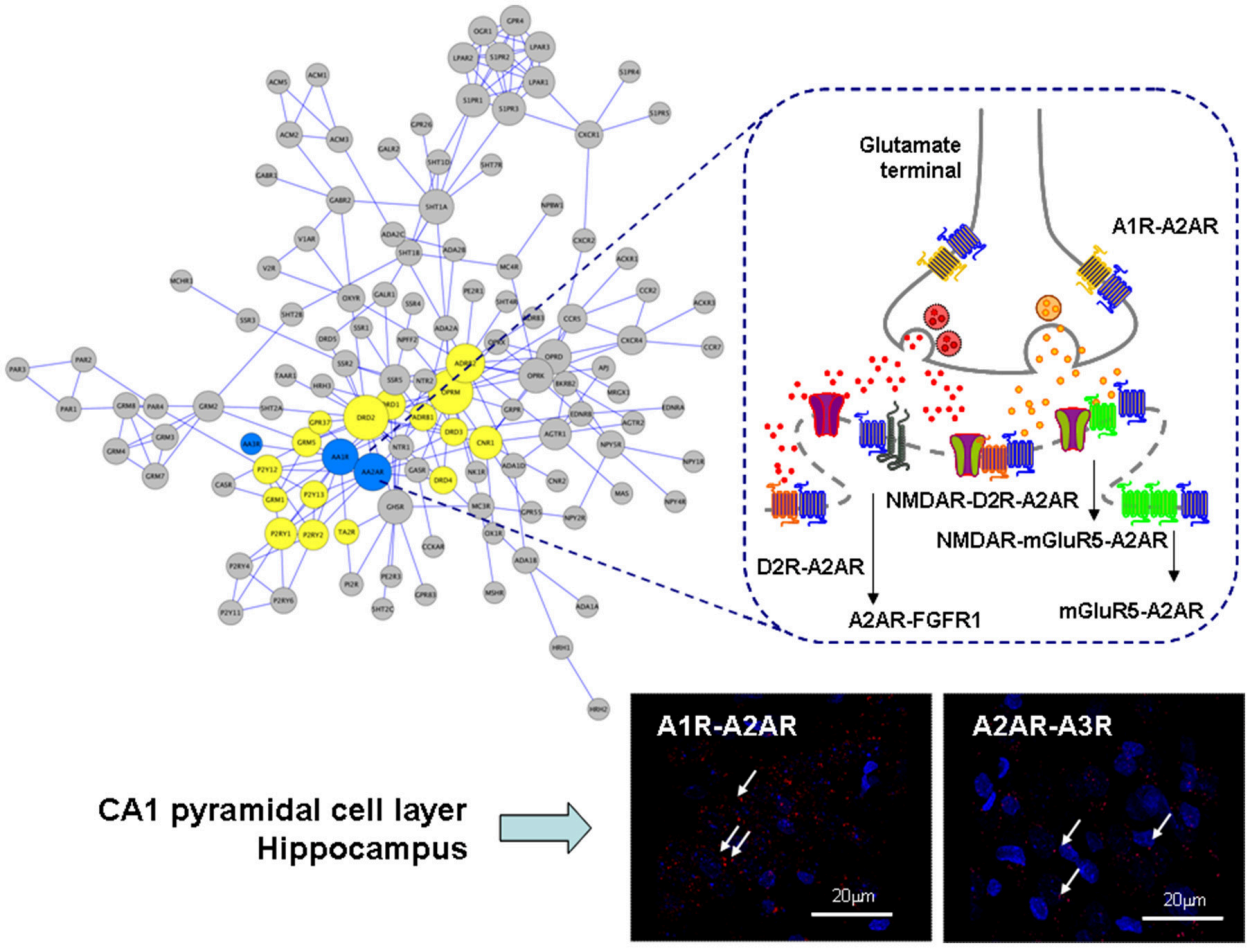
Illustration of the adenosine heteroreceptor complexes found in the GPCR heteroreceptor network (GPCR-hetnet; left large panel) and the synaptic and extrasynaptic regions of the glutamate synapse in the striato-pallidal GABA neurons (right panel). Also PLA positive red clusters of A1R-A2AR and A2AR-A3R isoreceptor complexes in the CA1 pyramidal cell layer (lower right panel) are shown (Lower right panels). In the GPCR-hetnet the adenosine isoreceptor complexes are highlighted in blue and adenosine heteroreceptor complexes in yellow. In the extrasynaptic regions the A1R-A2AR isoeceptor complex is shown in the glutamate nerve terminal and the A2AR-D2R and A2AR-mGluR5 at the postjunctional level. At the postsynaptic level the putative A2AR-mGluR5-NMDAR and A2A-D2R-NMDAR complexes are indicated together with A2AR-FGFR1. It seems likely that most heteroreceptor complexes can have both a synaptic and extrasynaptic position.

**Figure 2:**
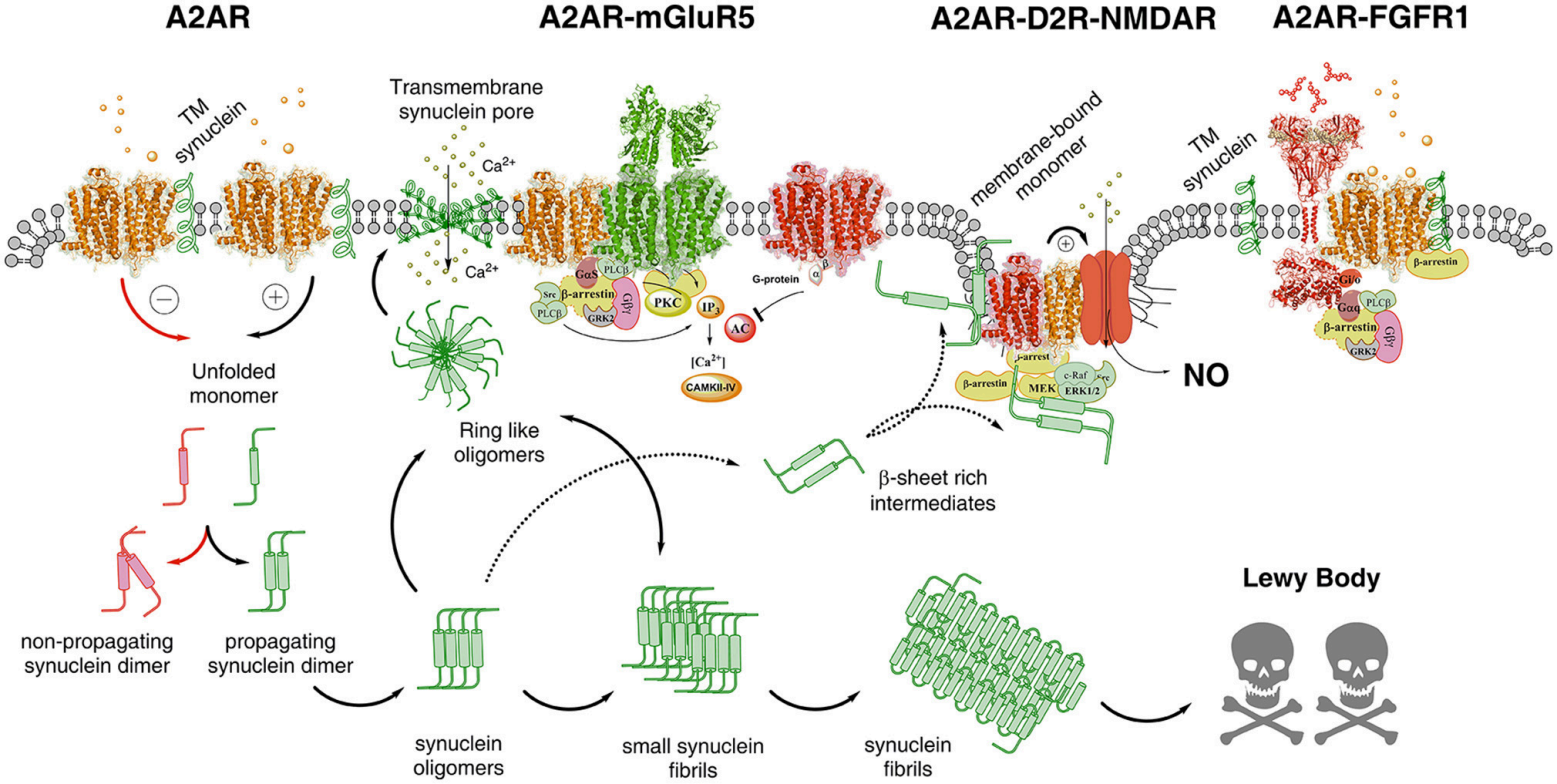
Illustration of possible molecular mechanism by which alpha-synuclein monomers/oligomers/ synuclein fibrils can modulate the homo-heteroreceptor complexes balance and panorama in the plasma membrane. In the far left part of the figure it is indicated that monomeric alpha-synuclein transmembrane (TM) peptide can become linked to A2AR homoreceptor complex and modulate the A2AR function. The A2AR antagonist may then favor the formation of non-propagating synuclein dimers (pathway highlighted in red). The A2A receptor agonist induced A2AR activation (pathway highlighted in green) may instead favor the propagation of the synuclein dimers/oligomers into small and large synuclein aggregates leading to formation of Lewy bodies. Ring-like synuclein oligomers may also be formed which may enter the membrane and there produce beta sheet structures that associate and give rise to pores in the plasma membrane through which calcium ions may pass. In the A2AR-mGluR5 heteroreceptor complex the signaling pathways are illustrated and how protein kinases like PKA, PKC and calcium-calmodulin kinase II activities can have a role in the modulation of the synuclein aggregation process. The A2AR-D2R-NMDAR complex, to which the alpha-synuclein monomer may bind to the A2AR, is also illustrated to indicate that A2AR activation can mediate toxicity also by turning on NMDAR signaling via inhibition of the D2R induced allosteric antagonistic interaction with the NMDAR. In this way the calcium influx through these ion channels is reduced as well as its coupling to nitric oxide (NO) toxicity. It is also indicated that beta sheet rich intermediates of alpha-synuclein peptides may bind to the intracellular loops and C-terminal of the receptor protomers of this heteroreceptor complex and modulate their signaling. They may also disturb the signaling of the G proteins and beta-arrestin. Finally to the far right the A2AR-FGFR1 heteroreceptor complex is presented with the alpha-synclein monomer bound to the A2AR. The role of this receptor complex in the degeneration process is unknown but FGFR1 activation by the A2AR may enhance structural plasticity and reduce toxicity.

The detailed mechanisms on how alpha-synuclein accumulation produces a marked increase of A2AR signaling leading to toxicity remain to be clarified but may involve increases in extracellular adenosine levels (Ferreira et al., 2017a) enhancing adenosine volume transmission events. It is proposed that alpha-synuclein monomers, especially in an alpha helix conformation, may bind to domains of the A2AR producing an enhanced Gs/olf coupling and/or modulation of A2A protomers in heteroreceptor complexes (Figures 1, 2). This proposal is based on the fact that GPCRs can bind to a number of proteins to form homo and heteroreceptor complexes (Bockaert et al., 2010; Fagni, 2012; Borroto-Escuela et al., 2016a). As a consequence PKA activity linked to the A2AR would be enhanced. It was in fact demonstrated that alpha-synuclein phosphorylation at serine 129 was associated with formation of Lewy bodies and neurodegeneration of DA neurons (Fujiwara et al., 2002). In line with these results A2AR antagonists can also reduce Tau hyperphosphorylation linked to memory improvement (Laurent et al., 2016). Such a scenario can help explain the neuroprotective actions of A2AR antagonists involving their ability to counteract the alpha-synuclein aggregation (Ferreira et al., 2017a). It also seems possible that the increased A2AR activation can bring down the activity of alpha-synuclein induced clearance via autophagy involving reduced lysosomal function (Decressac et al., 2013). Such events blocked by A2AR antagonists may lead to rescue of cells from alpha synuclein toxicity. Thus, the autophagy-lysosome pathway comes into focus.

In line with the view above, it was recently established that aberrant A2AR signaling can contribute to neurodegeneration and cognitive deficits in a model of synucleinopathy (Hu et al., 2016). Interestingly, A2AR deletion results in neuroprotection in a mouse model of tauopathy (Laurent et al., 2016). Neuroprotection was associated with reductions of Tau hyperphosphorylation and markers of neuroinflammation and counteractions of memory deficits. A2AR antagonists produced protective actions. Similar molecular mechanisms may be involved in the A2AR triggered molecular pathology as discussed above for alpha-synuclein. Indications were also found that GSK-3β, a serine-threonine kinase, and BDNF can participate in these events (Laurent et al., 2016). Taken together, these seminal papers make it clear that A2AR should become targets for treatment of alpha-synucleinopathies and Tauopathies.

### A2AR heteroreceptor complexes

#### Putative A2AR-D2R-NMDAR heteroreceptor complexes

The existence of A2AR-D2R heteroreceptor complexes with antagonistic A2AR-D2R interactions is well-known with A2AR activation inhibiting Gi/o mediated signaling of D2Rs (Hillion et al., 2002; Canals et al., 2003; Navarro et al., 2009, 2010; Borroto-Escuela et al., 2010a,b, 2013b; Figures 1, 2). They are highly expressed in the striato-pallidal GABA neurons in synaptic and extrasynaptic location related to glutamate synapses (Fuxe et al., 2005, 2010, 2014c,d). In these neurons also D2R-NMDAR heteroreceptor complexes exist in glutamatergic synapses with D2R activation leading to inhibition of NMDAR signaling (Liu et al., 2006). The interface involves the C-terminal part of the subunit GluN2B of the NMDAR and the intracellular loop 3 of the D2R. D2R activation interferes with the ability of CaMKII to bind to GluNR2B and therefore reduces the phosphorylation of this subunit leading to a reduction of the NMDAR currents (Liu et al., 2006).

Based on these results it seems possible that striatal A2AR-D2R-NMDAR heteroreceptor complexes may exist, in the striato-pallidal GABA neurons in a synaptic and/or extrasynaptic position, and in cortical regions but in lower densities than in the striatum. It may therefore be considered that alpha-synuclein induced altering of A2AR signaling can produce neuronal cell death through allosteric inhibition of D2R function which disinhibits NMDAR signaling in this heteroreceptor complex. As a result calcium influx is increased and excitotoxicity may develop.

It is known that calcium transients in spines can impact various signal transduction cascades leading inter alia to effects on cell survival and apoptosis, which are context dependent (Kennedy et al., 2005). It is of high interest that the C-terminal of the GluN2B subunit can be linked to the post-synaptic density 95 (PSD-95) protein via the PDZ domain (Kornau et al., 1995; Cui et al., 2007). Through this connection the activated NMDAR can become linked to nitric oxide neurotoxicity (Sattler et al., 1999). By counteracting this interaction through knockdown of PSD-95, calcium-activated nitric oxide production is blocked. The hypothesis of this trimeric heteroreceptor complex deserves to be tested in cellular models.

#### Putative A2AR-mGluR5-NMDAR heteroreceptor complexes

A2AR-mGluR5 heteroreceptor complexes were found in the striatum with synergistic receptor-receptor interactions located mainly in extrasynaptic regions (Ferre et al., 2002; Rodrigues et al., 2005; Tebano et al., 2005; Cabello et al., 2009; Borroto-Escuela et al., 2016a; Figure 1). Direct interactions were found between NMDAR and mGluR5 and reciprocal allosteric receptor-receptor interactions exist between them (Perroy et al., 2008). In neurons both synergistic and antagonistic receptor-receptor interactions can be observed between NMDAR and mGluR5 (Perroy et al., 2008). One explanation can be the dynamic association of the scaffolding proteins Homer-Shank to the mGluR5-NMDAR heteroreceptor complex (Husi et al., 2000). As examples can be mentioned that the D2 receptor can form a protein complex with Disrupted in Schizophrenia 1 (DISC1; Su et al., 2014) and with the sigma1 receptor (Borroto-Escuela et al., 2016b, 2017) altering its signaling and recognition in a dynamic way. Cocaine targets the sigma1 receptor with high affinity and modulates allosteric D2R-sigma1R receptor-receptor interactions on dopamine and glutamate nerve terminals from rat striatum (Beggiato et al., 2017). D2R-sigma1R-DAT complexes also appear to exist in the DA nerve terminals, in which cocaine in nanomolar concentrations increases D2R signaling.

In view of these findings there is the possibility that A2AR can increase NMDAR signaling also via allosteric enhancement of mGluR5 function. In this case, however, there is an allosteric enhancing action of NMDAR signaling produced by mGluR5. This proposal can be tested in neuronal cell cultures.

## Glial A2ARs and neuroinflammation. possible role of A2AR heteroreceptor complexes

### Microglia

It is important to underline that also adenosine A2ARs expressed in microglia can be involved in the neurodegenerative processes brought about by increased A2AR-mediated transmission. It is of high interest to note that one pathologic event is sufficient to induce a gain-of-function of microglial A2AR signaling via Gs-AC-PKA pathways but not of microglial adenosine A1R, A2BR, and A3R signaling (Santiago et al., 2014). This process involves enhancement of neuroinflammation through inter alia A2AR-induced expression and release of multiple proinflammaory cytokines like IL-1beta, IFN-gamma, and TNF from microglia (Santiago et al., 2014).

Through extracellular vesicle mediated volume transmission, proteins like receptors, and alpha-synuclein can be transferred from cell to cell via inter alia exosomes diffusing in the extracellular fluid to be incorporated into other cells through vesicular endocytosis (Agnati et al., 2010, 2014; Angot et al., 2012; Agnati and Fuxe, 2014; Borroto-Escuela et al., 2015; Dehay et al., 2016). This cell to cell transfer process of alpha-synuclein can explain the spread of alpha-synuclein induced neurodegeneration in the CNS and can involve both nerve cells, astroglia, and microglia (Borroto-Escuela et al., 2015; Fuxe and Borroto-Escuela, 2016). It seems possible that transfer of alpha-synuclein to microglia can be involved in the upregulation of microglial A2AR, which contributes to increased neuroinflammation.

The putative existence of A2AR isoreceptor complexes and their function in the microglia remains to be clarified. However, it has been shown that a receptor heteromer mediated regulation of endocannabinoid signaling exists in activated microglia. It is also of substantial interest that A2AR-CB1R-D2R heteroreceptor complexes were demonstrated in living cells using the sequential BRET-FRET technique (Carriba et al., 2008). Thus, A2AR-CB1R heteroreceptor complexes may exist in basal and/or activated microglia. There is an upregulation of CB1R-CB2R heteromers in activated microglia which is of relevance for Alzheimer's disease and levodopa-induced dyskinesia (Navarro et al., 2017). Dyskinesia in Parkinson's was found to correlate with CB1R-CB2R heteroreceptor complex upregulation in activated microglia. However, we also propose that the disbalance of activity in the direct and indirect pathways due to pathological alterations in the D1R and D2R heteroreceptor complexes in these two pathways can be a significant cause of dyskinesia development. Thus, e.g., the initiation of movements by the direct pathway may not be appropriately associated with a balanced reduction of motor inhibition of the indirect pathway. This may results in an exaggerated removal of motor inhibition which can lead to dyskinesia development upon treatment with L-DOPA.

It remains to be tested if also A2AR heteroreceptor complexes may participate in mediating proliferation and enhanced reactivity of upregulated microglia and have an impact on neuroinflammation and neurodegeneration. It is known that in neuroinflammation adenosine A2ARs can mediate microglial process retraction giving the microglia their ameboid appearance under these conditions (Orr et al., 2009). However, in another state of neuroinflammation A2AR activation can also trigger proliferation of microglia through microglial release of BDNF (Gomes et al., 2013) and/or nitric oxide release (Saura et al., 2005). Thus, the signaling consequences of A2AR activation in microglia may vary depending on the type of inflammatory state present in the microglia. Each state may be associated with the dynamic formation of special types of A2AR isoreceptor complexes dependent on the inflammatory state induced.

### Astroglia

Central dopamine and noradrenaline neurons communicate with astroglia via volume transmission and the existence of astroglial dopamine D1R and D2R receptors (Fuxe et al., 2015). The astroglial perivascular endfeet have an important role in the clearance of waste products via the Glymphatic system where G stands for glia (Iliff et al., 2013; Jessen et al., 2015). This function is related to their expression of a high density of aquaporin-4 (Aqp4) water channels reducing the resistance to water flow. It was proposed that GPCRs like A2AR and D2R can modulate the water influx and outflux over the Aqp4 channels (Fuxe et al., 2015) present in the plasma membrane of astroglia, via direct receptor-water channel interactions involving allosteric mechanisms.

Recently it was possible to demonstrate in astrocytes from adult striatum that D2R and A2AR receptors coexist in the same astrocyte process using confocal microscopy (Cervetto et al., 2017). Furthermore, A2AR activation was found to inhibit the D2R induced inhibition of astroglial glutamate release elicited by 4-aminopyridine (Cervetto et al., 2017). By itself the A2AR agonist lacked effects on the glutamate release from astroglia. This action suggests a receptor-receptor interaction in astroglial A2AR-D2R heteroreceptor complexes since it was blocked by a D2R synthetic peptide interfering with A2AR-D2R heteromerization (Cervetto et al., 2017). It seems possible that this receptor complex also can directly interact with the astroglial Aqp4 water channels.

Overactivity of astroglial A2AR would thus enhance astroglial glutamate release since there will be a brake on inhibitory astroglial D2R signaling via the A2AR protomer. The impact on synaptic glutamate transmission of increased astroglial glutamate release is difficult to foresee. Extrasynaptic mGluR should be mainly reached. If the major receptor activation by astroglial glutamate involves the inhibitory mGluR2-4, coupled to Gi/o and located on the glutamate nerve terminals, a reduction of neuronal glutamate release would take place with inhibition of glutamate transmission and reduction of the activity of the striato-pallidal GABA neurons (Kalivas, 2009; Kalivas et al., 2009). Thus, reduced toxicity will occur. However, if also extrasynaptic and postsynaptic mGluR1 and mGluR5 coupled to Gq are substantially activated by astroglial glutamate release, glutamate synaptic strength can be increased. This can involve increases in intracellular calcium levels and increased inhibition of D2R signaling in A2AR-D2R-mGlu5R complexes (Cabello et al., 2009), where A2AR and mGluR5 protomers synergize to inhibit D2R signaling mediated via Gi/o. Under such scenario, glutamate toxicity is not expected to change unless extrasynaptic NMDAR/AMPAR/ kainate receptors become critically activated by astroglial glutamate.

The astroglial glutamate transporter-1 is also of relevance for excitotoxicity. The mechanism involves Na/K-ATPase-alpha2 coupled to the astroglial glutamate transporter. It is of high interest that the A2AR can directly bind to the astroglial Na/K-ATPase-alpha2 as indicated using proximity ligation assay and co-immunoprecipitation (Matos et al., 2013). Furthermore, activation of the A2AR inhibits the astroglial glutamate uptake through inhibition of Na/K-ATPase-alpha2 activity. This antagonistic interaction involving the above described receptor-protein complexes can further increase the extracellular glutamate levels that may reach a critical level to produce excitotoxicity. Through this mechanism involving A2AR activation ion homeostasis as well as the astrocyte-neuron lactate shuttle requiring astroglial glutamate uptake can deteriorate (Pellerin et al., 1998). The shuttle means that there is a transfer of lactate from astrocytes to neurons. Thus, astrocytes serve as a source of lactate and neurons as a sink for lactate. In this way lactate can help glucose support oxidative metabolism in neurons made possible through astroglial processes (Pellerin et al., 1998).

## Formation of A2AR-A2BR heteroreceptor complexes provides a brake on A2AR recognition and signaling

It was recently found that the two isoreceptors A2AR and A2BR can form heteroreceptor complexes in living cells and in brain tissue using BRET, FRET, BiFC, and proximity ligation assay (PLA) techniques (Hinz et al., 2017, in press). Remarkably, the demonstration that the A2AR protomer within A2AR-A2BR heteroreceptor complex loses its high affinity binding coupled to marked reduction of the potency of A2AR agonists to activate the Gs-AC pathway with accumulation of cAMP (Hinz et al., 2017, in press). The A2BR is an adenosine receptor with a low affinity for adenosine in contrast to the A2AR with high affinity for adenosine. So far the A2AR-A2BR heterocomplexes have been found in the cerebral cortex and in the hippocampal cortex by using PLA (Hinz et al., 2017, in press).

Based on these exciting findings it seems possible that an upregulation of A2BR expression in the glial-neuronal networks of the brain, which have been reported in neuroinflammation (Feoktistov and Biaggioni, 2011), can block the neurodegenerative effects by A2AR inactivation. Such an upregulation may increase the formation of A2AR-A2BR heteroreceptor complexes which should markedly reduce the A2AR-mediated signaling. These events can take place when the two adenosine receptors are expressed in the same nerve or glial cells. However, it should be considered that through extracellular vesicle (ECV) mediated volume transmission (Guescini et al., 2012; Borroto-Escuela et al., 2015), A2BRs can be transferred to cells expressing A2AR and to form A2AR-A2BR heteroreceptor complexes. Recently, ECV-mediated intercellular communication in the CNS was classified as a component of VT (Borroto-Escuela et al., 2015). The concept of ECV-mediated VT was based on the highly significant work of Simons and Raposo (Simons and Raposo, 2009), which demonstrated the fundamental role of exosomes in intercellular communication. However, it should be underlined that also alterations in endogenous protein expression plays a major role in changing the protein panorama in the cells.

It is proposed that such processes with increased formation of A2AR-A2BR heteroreceptor complexes may represent an important mechanism to counteract the neurodegenerative and toxic effects in models of brain disease of the expression and *in vitro* activation of A2ARs (as discussed above). Targeting and increasing expression of A2AR-A2BR heteroreceptor complexes via gene therapy involving the A2BR gene or a pharmacological approach may be a novel strategy for treatment of neurodegenerative disease in which A2AR over activation and/or overexpression plays a detrimental role. The pharmacological analysis can involve the identification of the transcription factors activating the expression of the A2BR as well as the use of a brain-penetrant heterobivalent compound built up of an A2A receptor antagonist pharmacophor and an A2BR agonist pharmacophor. Such a heterobivalent compound may assist in the heteromerization process. The A2AR-A2BR heteromer may be in balance with A2AR and A2BR homomers and other types of A2AR and A2BR heteroreceptor complexes. This homo and heteroreceptor panorama may vary among brain regions and within glial-neuronal networks of the same region and also among individual neurons and different types of glial cells in the same region.

It should be mentioned that A2BRs can also counteract the A1R produced inhibition of the glutamatergic transmission of the hippocampus (Goncalves et al., 2015). It seems likely that these findings reflect the existence of A1R-A2BR heteroreceptor complexes in glutamate nerve terminals, where the two adenosine receptor subtypes seem to be coexpressed.

## On the role of A1R-A2AR heteroreceptor complexes

In line with the above proposal is the early demonstration that A1R-A2AR heteromers exist in striatal and hippocampal glutamate nerve terminals where A1R and A2AR receptors were found to be colocalized (Rebola et al., 2005) and form an isoreceptor complex in the rat brain (Figure 1). The major receptor-receptor interaction found appears to be an A2AR agonist produced reduction of A1R affinity. Therefore, at high concentrations of adenosine, which can activate A2ARs, an increase of glutamate release is found. In astrocytes a similar mechanism maybe found involving A1R-A2AR heteromers which via Gi/o and Gs proteins modulate GABA transport (Cristovao-Ferreira et al., 2011, 2013). It was proposed that the structure might be based on heteromers formed from homomers (Navarro et al., 2016b).

Taken together, it seems possible that with increased concentrations of adenosine the allosteric receptor-receptor interactions in the A1R-A2AR and putative A1R-A2BR isoreceptor complexes will favor glutamate release which may contribute to enhanced excitation and possible excitotoxicity.

## On the existence of adenosine A2AR-receptor tyrosine kinase heteroreceptor complexes and their role in neuroprotection

As clearly pointed out by Gomes et al. (2011), it is still not clear if the trophic factors and their tyrosine kinase receptors (RTK) play a significant role in the regulatory actions of adenosine via A2AR activation, since these signals are supposed to produce neuroprotection. A2AR receptor antagonists in fact counteract neurodegeneration in a number of models. Nevertheless, there is one interesting and significant publication that demonstrates that activation of A2AR receptors can enhance Trk neurotrophin receptor signaling which in this preparation developed independently of neuroptrophins (Lee and Chao, 2001). Furthermore, in a beautiful paper FGF2 and A2AR agonists were found to act via FGFR1 and A2AR to enhance synaptic plasticity in the striatum (Flajolet et al., 2008). In this case an FGFR1-A2AR heteroreceptor complex was demonstrated (Flajolet et al., 2008; Borroto-Escuela et al., 2013a; Figure 2). It seems likely that the results obtained in the Lee and Chao paper (Lee and Chao, 2001) also reflects the existence of heteroreceptor complexes between TrkA and A2AR and TrkB and A2AR in which A2ARs enhance Trk signaling.

In line with these findings it was also found that chronic *in vivo* treatment with an A2AR antagonist blocked the BDNF induced facilitation of LTP. Thus, a reduction of BDNF action was achieved (Jeronimo-Santos et al., 2014).

Based on the work presented in this article it appears that treatment with A2AR antagonists and increased formation of A2AR-A2BR heteroreceptor complexes should represent novel strategies for alpha-synyclein induced neurodegeneration. However, in the case of deficits in neurotransmission plasticity A2AR agonists may be the way to go for treatment in view of their ability to enhance TRK signaling.

## Functional A2AR-glucocorticoid receptor interactions

It was recently demonstrated that A2ARs can induce a dysfunction of the hypothalamic-pituitary-adrenal (HPA) axis by targeting the function of glucocorticoid receptors (GR; Batalha et al., 2016). Overactivation of A2ARs leads to loss of plasma glucocorticoid levels and reduction of hippocampal levels of GR. The A2AR activation affected GR function by modulating the transcriptional activity of GR and their nuclear location. These are exciting findings and show that the A2AR is a major modulator of the function of GR. It can represent one important mechanism for the cognitive enhancement with improvement of memory produced by A2AR antagonists (Batalha et al., 2016).

In our opinion a direct interaction of the membrane located A2ARs with cytosolic-perimembrane GR should also be considered. Glucocorticoid receptors also exist at the neuronal membrane and can regulate nongenomic corticosteroid signaling (Groeneweg et al., 2012). Thus, a direct receptor-receptor interaction between the intracellular domains of the A2AR and GR becomes possible. Through the formation of this complex the A2AR can modulate the function of GR in two ways. The first possibility is that the A2AR-GR complex became recruit to the lysosome. As a results only the intracellular domains of the A2AR may remain linked to the GR and be translocated to the nucleus as a new component of GR. The link of these A2AR fragments to the GR element can substantially alter the GR transcriptional activity and represent a novel mechanism for modulation of gene transcription. Another alternative is the A2AR is available to recruit GR to the plasma membrane to an increased extent. In this way less GR will be available for translocation into the nucleus. In this way the transcriptional activity of GR can become reduced, instead the nongenomic GR signaling become dominant.

Interestingly, recent work demonstrated that the GR binds to GREs in β*-arrestin 1* and *2* and modulates their gene expression (Oakley et al., 2012) which will became reduced by the A2AR-GR heteroreceptor complex. Such mechanisms may in fact have a major impact on the GR function leading to a marked disturbance of synaptic plasticity and memory. Thus, it is proposed that such a A2AR-GR heteroreceptor complex may lead to a mechanism that can change the hippocampal transcription panorama. The hippocampal connection to the hypothalamus may also be affected leading to hypothalamic-pituitary-adrenal dysfunction. Future work will establish if this hypothesis is of value.

We have previously introduced the theory that long-term memory becomes possible by the translocation of parts of the heteroreceptor complexes into the nucleus over the perinuclear membrane and promote the activation capability of a particular set of transcription factors (Fuxe et al., 2014a,b; Borroto-Escuela et al., 2015). These may then induce the transcription of unique adaptor proteins that upon expression can bind to the heteroreceptor complexes in the cytoplasmic component of the plasma membrane and link them to the cytoskeleton and major scaffolding proteins (Fuxe et al., 2014b). In this way they become stabilized with conserved allosteric receptor-receptor interactions.

## A3R heteroreceptor receptor complexes

It should be noted that also adenosine A3Rs take part in heteroreceptor complexes. So far A1R-A3R complexes have been demonstrated in the cerebral cortex and dorsal hippocampus (Hill et al., 2014) and A2AR-A3R isoreceptor complexes in the dorsal hippocampus mainly in the pyramidal cell layer using proximity ligation assay (Borroto-Escuela et al., 2016a; Figure 1). The function of complexes including A3R has not yet been clarified.

## Conclusions

The exciting work performed on A2AR mediated neurodegeneration performed by several groups (Gomes et al., 2011; Santiago et al., 2014; Laurent et al., 2016; Ferreira et al., 2017a) opens up a major role for A2AR antagonists in neurodegenerative disease involving alpha-synucleins. In this perspective article we have proposed that adenosine A2AR iso- and heteroreceptor complexes and their allosteric receptor-receptor interactions can be key players in balance with corresponding homorecepor complexes. In future work it will therefore be crucial to characterize the heteroreceptor complexes with FRET/BRET and proximity ligation assay and their allosteric receptor-receptor interactions with radioligand binding assays and their signaling pathways with luciferase reporter gene assays. The receptor interface is usually different between a receptor heteromer and the corresponding homomers. Therefore, it becomes possible to selectively interfere with formation of a receptor heteromer and thus its function vs. corresponding homomers through use of interface interacting peptides (Borroto-Escuela et al., 2010b, 2018).

Also a scheme was introduced how alpha-synuclein monomers/oligomers/ synuclein fibrils can modulate the adenosine A2AR homo-heteroreceptor panorama in the plasma membrane through intramembrane and intracellular interactions enhancing neurodegeneration (Figure 2). They can exist both in neurons, astroglia and microglia. The role of putative A2AR-D2R-NMDAR and A2AR-mGluR5-NMDAR heteroreceptor complexes were inter alia discussed in relation to glutamate synapses and excitotoxicity. Future work is necessary to test the impact of these adenosine A2AR-containing complexes on A2AR mediated neurodegeneration, especially when mediated by alpha-synucleins.

## Author contributions

We confirm and declare that all authors meet the criteria for authorship according to the ICMJE, including approval of the final manuscript, and they take public responsibility for the work and have full confidence in the accuracy and integrity of the work of other group authors. They have substantially contributed to the conception or design of the work. Also they have also helped revising it critically for important intellectual content; and final approval of the version to be published. In addition, they have contributed in this last version of the manuscript in writing assistance, technical editing, language editing, and proofreading.

### Conflict of interest statement

The authors declare that the research was conducted in the absence of any commercial or financial relationships that could be construed as a potential conflict of interest.
